# *KRAS* Mutations Predict Inferior Post‐Remission Outcomes in Newly Diagnosed Acute Myeloid Leukemia With MAPK Pathway Mutations

**DOI:** 10.1111/ejh.70270

**Published:** 2026-07-24

**Authors:** Kensuke Takaoka, Sarah Mott, Ratdanai Yodsuwan, Sakditad Saowapa, Manasawee Tanariyakul, Chalothorn Wannaphut, Kittika Poonsombudlert

**Affiliations:** ^1^ Department of Internal Medicine University of Iowa Health Care Iowa City Iowa USA; ^2^ University of Iowa Health Care Holden Comprehensive Cancer Center Iowa City Iowa USA; ^3^ Department of Medicine University of Hawai'i John A. Burns School of Medicine Honolulu Hawaii USA

**Keywords:** AML, CRc, HSCT, KRAS, MAPK, NRAS, OS, RFS

## Abstract

Somatic mutations in the MAPK signaling pathway are frequently identified at AML diagnosis; however, their impact on treatment resistance and long‐term survival remains unclear. We conducted a retrospective study of patients with newly diagnosed MAPK‐mutated AML seen at the University of Iowa Health Care. The endpoints were overall survival (OS), composite complete remission (CRc) following induction therapy, and relapse‐free survival (RFS). Logistic and Cox regression models were used. Seventy‐four patients were included and 62 were induction eligible. Of the 62 patients receiving treatment, intensive induction was administered in 52.7%. The estimated 5‐year OS was 28.9%. On multivariable analysis, European LeukemiaNet 2022 adverse risk (hazard ratio [HR]: 3.56, 95% confident interval [CI]: 1.52–8.32) and *KRAS* mutation (HR: 2.14, 95% CI: 1.10–4.17) conferred inferior OS. Abnormal karyotype (odds ratio [OR]: 0.25, 95% CI: 0.07–0.89) and increasing age (OR: 0.97, 95% CI: 0.94–0.99) were associated with decreased odds of achieving a CRc. The median RFS after achieving a CRc was 13.3 months. We found that *KRAS* mutation conferred an inferior effect on OS and RFS but not CRc. These findings suggest that *KRAS* mutations may confer adverse prognostic significance and warrant validation in larger, multicenter cohorts.

## 1 Introduction

Acute myeloid leukemia (AML) is a biologically heterogeneous hematologic malignancy characterized by diverse cytogenetic and molecular abnormalities [1]. Somatic mutations in the MAPK signaling pathway, including *NRAS*, *KRAS*, *HRAS*, and *BRAF*, are frequently detected at the time of AML diagnosis [2, 3, 4]. The MAPK pathway plays a central role in regulating cellular proliferation, differentiation, and survival, and its dysregulation has been implicated in leukemogenesis, disease progression, and relapse [4]. Although European LeukemiaNet (ELN) 2024 genetic risk classification demonstrated adverse prognostic implications of *KRAS* mutations in AML patients treated with less‐intensive regimens, prior studies evaluating the prognostic impact of MAPK pathway mutations in AML survival outcomes of AML patients have been inconsistent [2, 3, 5, 6, 7].

The treatment strategies for AML patients in 2026 vary based on: cytogenetic and molecular drivers as well as age, comorbidities, and fitness. Older or medically unfit patients increasingly receive nonintensive regimens (NIR) such as hypomethylating agents (HMAs) with or without venetoclax, whereas younger fit patients are more likely to undergo intensive induction regimen (IIR) followed by allogeneic hematopoietic stem cell transplant (HSCT), if indicated [8, 9]. How the MAPK pathway mutations influence response to different treatment intensities, achievement of complete remission, relapse‐free survival (RFS), and long‐term survival in real‐world clinical practice remains incompletely characterized. To address these knowledge gaps, we conducted a retrospective cohort study of patients with newly diagnosed MAPK‐mutated AML treated at our center, and evaluated their clinical characteristics, treatment responses, and long‐term outcomes.

## 2 Methods

### 2.1 Patients

We conducted a retrospective analysis of patients with newly diagnosed AML harboring MAPK pathway mutations including: *NRAS*, *KRAS*, *HRAS*, and *BRAF* seen at the University of Iowa Health Care (UIHC) between December 2015 and March 2024. This study was approved by the University of Iowa Institutional Review Board.

### 2.2 Patient Population

Patients were adults aged 18 years and older diagnosed with AML per WHO 2022 criteria [10]. MAPK mutations were detected from the UIHC institutional next‐generation sequencing (NGS) panel, which uses a massively parallel NGS‐by‐synthesis (Ion Torrent)‐based assay that targets 36 AML‐related genes (list of genes provided in Table S1). Identified mutations were categorized using guidelines established by the Association for Molecular Pathology on interpreting somatic sequence variants, and the threshold for reporting a mutation is variant allelic frequency (VAF) ≥ 1% (details of MAPK mutation variants are available in Table S2). Minimal residual disease (MRD) after induction therapy was evaluated by multiparametric flow cytometry.

IIR includes the backbone of high‐dose chemotherapy such as 7 + 3 (anthracycline/cytarabine or FLAG‐Ida [fludarabine/cytarabine/G‐CSF/Idarubicin]) while NIR includes HMAs with or without venetoclax. Some patients who are deemed unfit for AML‐directed therapy received no treatment. Treatment response was defined according to the response criteria in AML of the ELN 2022 [11]. Among those who underwent HSCT, the conditioning regimen was classified as myeloablative conditioning (MAC) when it included fractionated total body irradiation > 8 Gray or intravenous busulfan ≥ 9 mg/kg; otherwise, it was classified as reduced‐intensity conditioning (RIC) [12]. The most common graft‐versus‐host disease (GVHD) prophylaxis was posttransplant cyclophosphamide with tacrolimus and mycophenolate mofetil.

### 2.3 Endpoints

The endpoints were overall survival (OS), composite complete remission (CRc) following induction therapy, defined as complete remission (CR), CR with incomplete hematologic recovery (CRi), or CR with partial hematologic recovery (CRh) by the ELN 2022 [11]. RFS was also evaluated among patients who achieved CRc (*n* = 33).

### 2.4 Statistical Analysis

Chi‐squared or Fisher's exact tests were used to compare categorical variables, and Wilcoxon rank sum tests were used to compare continuous variables between treatment groups. Logistic regression models were utilized to estimate the effect of patient, disease, and treatment characteristics on the odds of achieving a CRc among patients who received treatment and receipt of HSCT. *KRAS* and *NRAS* concurrent mutations were evaluated separately. Survival probabilities were estimated and plotted using the Kaplan–Meier method [13]. Time was calculated from diagnosis to death due to any cause for OS. Otherwise, patients were censored at last contact. Achieving a CRc and receipt of HSCT were included as time‐dependent covariates. For RFS, time was calculated from CRc to relapse or death due to any cause. Otherwise, patients were censored at HSCT or last disease evaluation. Cox regression models were used to estimate the effect of patient, disease, and treatment characteristics on OS and RFS. All statistical testing was two‐sided and assessed for significance at the 5% level using SAS v9.4 (SAS Institute, Cary, NC).

## 3 Results

### 3.1 Patient Characteristics

A total of 74 patients were included in the analysis. The median age at diagnosis was 66 years (range: 20–90 years), and 26 patients (35.1%) were female. Sixty‐six patients (90.4%) were White, with four patients (5.5%) identifying as Hispanic. The median absolute neutrophil count at diagnosis was 2.1 × 10^9^, and the median bone marrow blast percentage was 52%. At diagnosis, 28 patients (40.6%) had a normal karyotype, and 44 patients (61.1%) were categorized as adverse risk according to the ELN 2022 risk classification, followed by 17 (23.6%) as favorable risk. ELN risk classification was unavailable for two patients (Table 1).

**TABLE 1:** Patient characteristics by induction treatment.

No. (%)/median (range)	Total *N* = 74	Intensive *N* = 39	Nonintensive *N* = 23	No treatment *N* = 12	*p*
Age at diagnosis (years)	66 (20–90)	52 (20–81)	73 (53–90)	74 (45–89)	**< 0.01**
Sex					0.29
Female	26 (35.1)	11 (28.2)	11 (47.8)	4 (33.3)	
Male	48 (64.9)	28 (71.8)	12 (52.2)	8 (66.7)	
Race/ethnicity					0.50
Black	3 (4.1)	2 (5.1)	0	1 (9.1)	
Hispanic	4 (5.5)	2 (5.1)	1 (4.3)	1 (9.1)	
White	66 (90.4)	35 (89.7)	22 (95.7)	9 (81.8)	
Absolute neutrophil count (×10^9^/L) at diagnosis	2.1 (0.0–59.9)	1.9 (0.0–39.1)	2.0 (0.0–29.5)	6.0 (0.0–59.9)	0.20
Bone marrow blast count (%) at diagnosis	52 (20–99)	57 (22–96)	40 (20–99)	51 (24–86)	0.56
Karyotype					0.10
Normal	28 (40.6)	16 (44.4)	11 (47.8)	1 (10.0)	
Other	41 (59.4)	20 (55.6)	12 (52.2)	9 (90.0)	
ELN 2022 risk					0.38
Adverse	44 (61.1)	22 (56.4)	13 (56.5)	9 (90.0)	
Intermediate	11 (15.3)	6 (15.4)	5 (21.7)	0	
Favorable	17 (23.6)	11 (28.2)	5 (21.7)	1 (10.0)	
MAPK mutation^a^					0.72
NRAS	50 (71.4)	27 (75.0)	15 (65.2)	8 (72.7)	0.66
KRAS	24 (32.4)	12 (30.8)	9 (39.1)	3 (25.0)	0.64
BRAF	4 (5.5)	2 (5.3)	2 (8.7)	0	0.16
HRAS	1 (1.4)	0	0	1 (8.3)	
CRc after induction Tx					0.51
Yes	33 (56.9)	22 (57.9)	11 (55.0)	—	
No	25 (43.1)	16 (42.1)	9 (45.0)	—	
HSCT					**< 0.01**
Yes	22 (35.5)	20 (51.3)	2 (8.7)	—	
No	40 (64.5)	19 (48.7)	21 (91.3)	—	
Conditioning regimen					1.00
MAC	15 (68.2)	14 (70.0)	1 (50.0)	—	
RIC	7 (31.8)	6 (30.0)	1 (50.0)	—	

*Note:* Bold indicates statistically significant values.

Abbreviations: CRc, composite complete remission; ELN, European LeukemiaNet; HSCT, hematopoietic stem cell transplant; MAC, myeloablative conditioning; RIC, reduced‐Intensity conditioning; Tx, treatment.

^a^ MAPK mutation status was unavailable in four patients with *NRAS*, one patient with *BRAF*, and *HRAS* due to incomplete NGS data.

### 3.2 NGS

MAPK pathway mutations were distributed as follows: *NRAS* only (*n* = 45), *KRAS* only (*n* = 19), concurrent *NRAS*/*KRAS* mutations (*n* = 5), *BRAF* mutations (*n* = 4), and *HRAS* mutation (*n* = 1) (Figure 1). The most common variant was G12D, observed in both *KRAS* (6 cases, 25.0%) and *NRAS* (14 cases, 28.0%). In *NRAS*, the second most frequent mutation was G13D (10 cases, 20.0%), followed by Q61H (8 cases, 16.0%), and Q61K (8 cases, 16.0%). Among *BRAF*‐mutated cases, three of four (75.0%) harbored the V600E variant. The single *HRAS*‐mutated case (patient #67) carried a frameshift mutation (H27fs). The most frequent double co‐mutation pairs were *NPM1*/*NRAS* (*n* = 13), followed by *KRAS*/*TET2* (*n* = 12), and *SRSF2*/*TET2* (*n* = 12). *TP53* co‐mutations were observed in five cases, including three with *NRAS* mutations, one with a *KRAS* mutation, and one with an *HRAS* mutation. The detailed NGS results are provided in Table S3.

**FIGURE 1:**
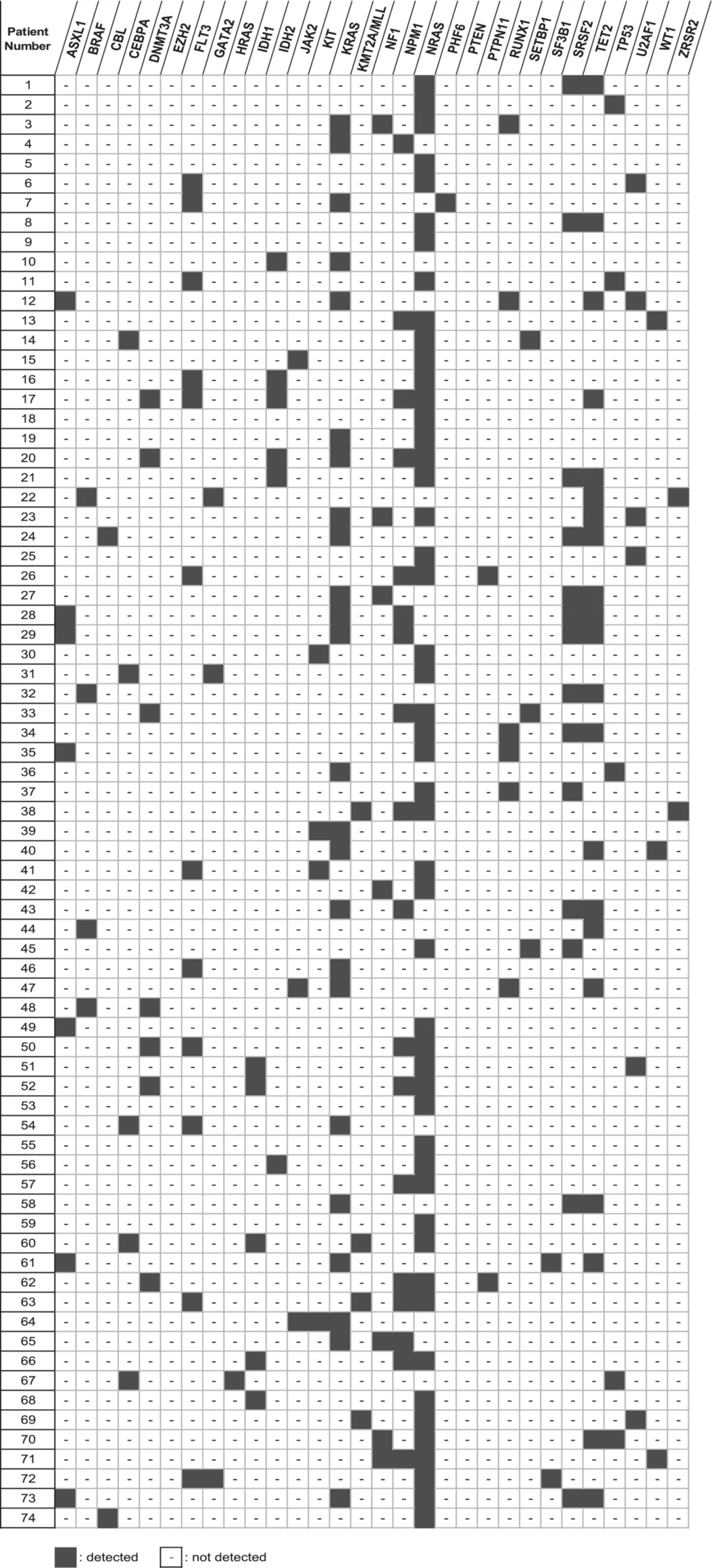
Next‐generation sequencing results of the entire cohort (*n* = 74).

### 3.3 Response to Therapy

Among treated patients (*n* = 62), 39 patients (52.7%; median age, 52 years) received IIR, while 23 patients received NIR (median age, 73 years). The CRc rates were 57.9% in the IIR group (22 of 38 evaluable patients; one patient had missing response data) and 55.0% in the NIR group (11 of 20 evaluable patients; three patients had missing response data). Among the 23 patients who received NIR, 22 received HMAs (azacitidine‐based *n* = 13, decitabine‐based *n* = 9) with a CRc rate of 30.8% and 77.8% for azacitidine‐based and decitabine‐based therapy, respectively (Table S4). Among the 33 patients who achieved CRc following induction therapy, 11 were MRD‐positive and 20 were MRD‐negative, while MRD status was unavailable for 2 patients. On univariable analysis, a normal karyotype was significantly associated with higher odds of achieving a CRc (odds ratio [OR]: 3.47), whereas ELN 2022 adverse‐risk classification was associated with lower odds of CRc (OR: 0.21) (Table 2). In multivariable analysis, karyotype (*p* = 0.03) and age (*p* < 0.05) remained independently associated with the odds of achieving CRc. Patients with a normal karyotype had a nearly fourfold increased odds of achieving CRc (OR: 3.95; 95% CI: 1.12–13.95). Increasing age was associated with reduced odds of CRc (OR per year, 0.97; 95% CI: 0.94–0.99). In contrast, ELN 2022 adverse‐risk classification was no longer significantly associated with CRc after adjustment.

**TABLE 2:** Univariable and multivariable logistic regression for achieving a composite complete remission in treated patients (*n* = 62).

	Univariable			Multivariable		
	OR	95% CI	*p*	OR	95% CI	*p*
Age (unit = 1)	0.98	0.95–1.00	0.07	0.97	0.94–0.99	**< 0.05**
Sex		0.38–3.08	0.88			
Female	1.09					
Male	Ref					
Karyotype		1.17–10.29	**0.02**		1.12–13.95	**0.03**
Normal	3.47			3.95		
Other	Ref			Ref		
ELN 2022 risk		0.07–0.62	**< 0.01**		0.12–1.30	0.12
Adverse	0.21			0.39		
Other	Ref			Ref		
MAPK mutation						
NRAS						
Yes	1.9	0.61–5.97	0.27			
No	Ref					
KRAS						
Yes	0.71	0.25–2.04	0.53			
No	Ref					
VAF (%)						
NRAS (units = 1%)	1.01	0.97–1.06	0.49			
KRAS (units = 1%)	1.01	0.96–1.06	0.78			
Induction treatment		0.50–3.97	0.51			
Intensive	1.41					
Nonintensive	Ref					

*Note:* Bold indicates statistically significant values.

Abbreviations: CI, confidence interval; ELN, European LeukemiaNet; OR, odds ratio; Ref, reference; VAF, variant allele frequency.

### 3.4 Survival Analysis

With a median follow‐up of 7.9 months, 49 patients had died in the study at the time of data cut off. Of the 33 patients who achieved CRc, 31 patients had follow‐up after CRc, 12 patients relapsed, and 6 patients experienced non‐relapse mortality. The median survival was 10.9 months with a five‐year OS of 28.9% (95% CI: 17.7%–41.1%) (Figure 2A). Median OS for IIR, NIR, and no treatment were 58.7, 7.5, and 0.2 months, respectively (Figure 2B). The median OS among all patients with MAPK‐mutated AML was 17.8 months (95% CI: 7.7–30.3 months). Among patients with available MRD assessment after achieving CRc, the median OS was 57.8 months (95% CI: 14.6–not reached) in MRD‐negative patients and 24.9 months (95% CI: 2.6–not reached) in MRD‐positive patients. On univariate analysis among treated patients (*n* = 62), increasing age (hazard ratio [HR] per year, 1.04; 95% CI: 1.02–1.06; *p* < 0.01), ELN 2022 adverse risk (HR: 4.05; 95% CI: 1.92–8.53; *p* < 0.01), absence of an *NRAS* mutation (HR: 2.81; 95% CI: 1.45–5.44; *p* < 0.01), presence of a *KRAS* mutation (HR: 2.92; 95% CI: 1.52–5.59; *p* < 0.01), receipt of NIR (HR: 2.81; 95% CI: 1.46–5.44; *p* < 0.01), and failure to achieve CRc (HR: 2.16; 95% CI: 1.08–4.33; *p* = 0.03) were each associated with inferior OS. In multivariable analysis, ELN 2022 adverse risk (HR: 3.56; 95% CI: 1.52–8.32; *p* < 0.01) and *KRAS* mutation (HR: 2.14; 95% CI: 1.10–4.17; *p* = 0.03) remained independently associated with inferior OS, whereas age and induction intensity were no longer statistically significant (Table 3). The median RFS after achieving a CRc was 13.3 months (95% CI: 6.9–24.9), with a 3‐year RFS of 28.0% (95% CI: 15.4–51.2) (Figure 3). Among patients with available MRD assessment after achieving CRc, the median RFS was 14.6 months (95% CI: 6.9–not reached) in MRD‐negative patients and 18.3 months (95% CI: 0.4–not reached) in MRD‐positive patients. On multivariable analysis, *KRAS* mutation (HR: 9.51, 95% CI: 2.20–41.08) remained significantly associated with poorer RFS, whereas treatment intensity did not (Table S5).

**FIGURE 2:**
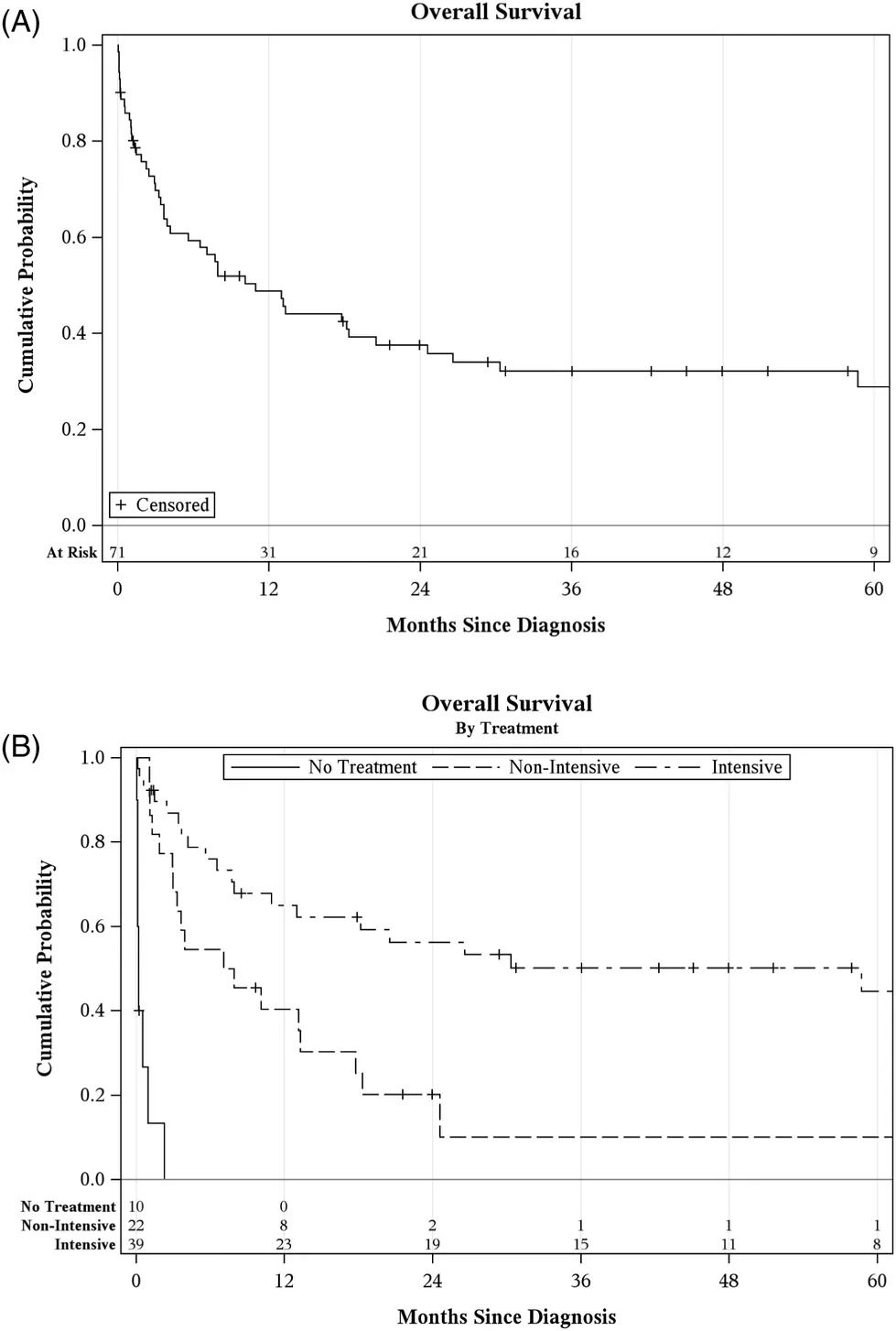
(A) Overall survival of the entire cohort (*n* = 71); (B) Overall survival by induction treatment (*n* = 71).

**TABLE 3:** Univariable and multivariable Cox regression analyses of overall survival in treated patients (*n* = 61).

	Univariable			Multivariable		
	HR	95% CI	*p*	HR	95% CI	*p*
Age (unit = 1)	1.04	1.02–1.06	**< 0.01**	1.02	1.00–1.05	0.11
Sex		0.61–2.29	0.62			
Female	1.18					
Male	Ref					
Karyotype		0.72–2.74	0.31			
Other	1.41					
Normal	Ref					
ELN 2022 Risk		1.92–8.53	**< 0.01**		1.52–8.32	**< 0.01**
Adverse	4.05			3.56		
Other	Ref			Ref		
MAPK mutation						
NRAS						
No	2.81	1.45–5.44	**< 0.01**			
Yes	Ref					
KRAS						
Yes	2.92	1.52–5.59	**< 0.01**	2.14	1.10–4.17	**0.03**
No	Ref			Ref		
VAF (%)						
NRAS (units = 1%)	0.99	0.97–1.02	0.62			
KRAS (units = 1%)	1.02	0.99–1.04	0.16			
Induction treatment			**< 0.01**			0.11
Non‐intensive	2.81	1.46–5.44		2.06	0.84–5.04	
Intensive	Ref			Ref		
Achieving CRc			**0.03**			
No	2.16					
Yes	Ref					
MRD after induction treatment		0.43–3.92	0.64			
Positive	1.30					
Negative	Ref					
HSCT		0.93–6.50	0.07			
No	2.46					
Yes	Ref					

*Note:* Bold indicates statistically significant values.

Abbreviations: CI, confidence interval; CRc, composite complete remission; ELN, European LeukemiaNet; HR, hazard ratio; HSCT, hematopoietic stem cell transplant; MRD, minimal residual disease; Ref, reference.

**FIGURE 3:**
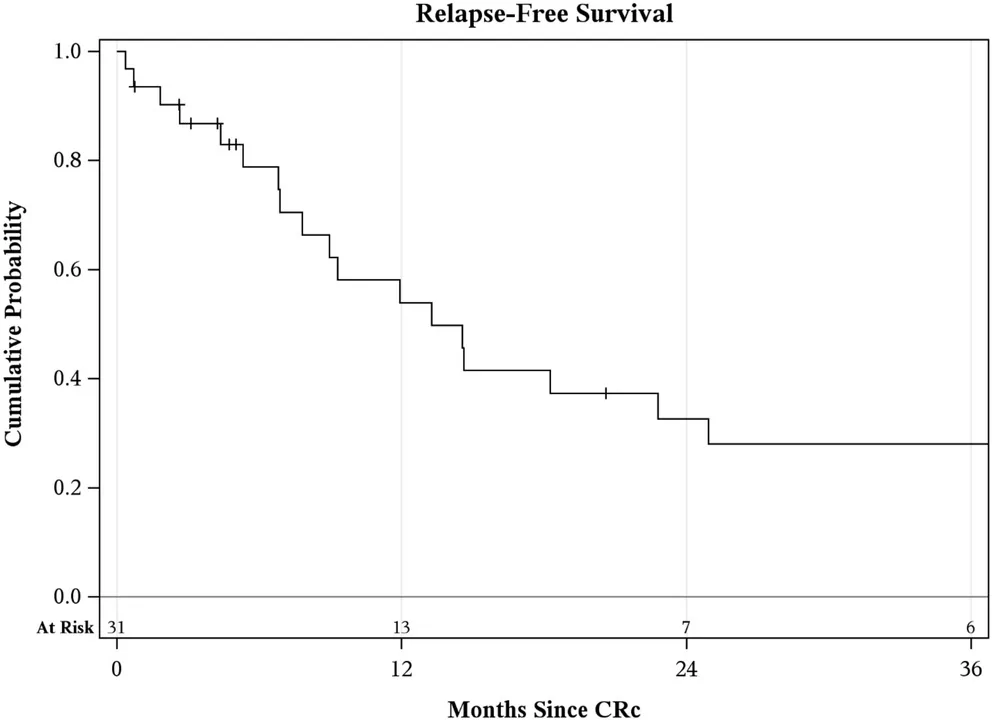
Relapse‐free survival in patients achieving composite complete remission (*n* = 31).

### 3.5 Allogeneic HSCT

Twenty‐two patients underwent HSCT, and 15 (68.2%) received MAC. Sixteen patients (72.7%) underwent HSCT in first CR, five (22.7%) in second CR, and one (4.5%) with primary refractory disease. Posttransplant relapse occurred in 11 patients, including seven who received MAC and three who received RIC. Among treated patients (*n* = 62), univariable analysis showed that increasing age was associated with decreased odds of undergoing HSCT (OR: 0.92), whereas receipt of IIR was associated with increased odds of HSCT (OR: 11.05). On multivariable analysis, increasing age remained independently associated with lower odds of allogeneic HSCT (OR: 0.93; 95% CI: 0.89–0.97; *p* < 0.01), whereas treatment intensity was no longer significantly associated with receipt of HSCT (Table S6).

## 4 Discussion

In this real‐world retrospective cohort study, we demonstrated that *KRAS* mutations are independently associated with inferior OS, even after adjustment for age, ELN 2022 risk classification, and treatment intensity. Although *KRAS* mutations were not associated with the likelihood of achieving CRc, they were independently associated with inferior RFS among patients achieving remission. Together, these findings suggest that the adverse prognostic impact of *KRAS* mutations in AML may not be driven by failure to achieve remission, but rather by reduced durability of remission and adverse post‐remission disease biology.

Notably, *KRAS* mutation was independently associated with shorter RFS among patients who achieved CRc. However, from the statistical perspective, OS was calculated from the entire cohort, while RFS only included those who have achieved CRc; therefore, the degree of statistical significance should not be directly compared. Nevertheless, the observed association between *KRAS* mutation and inferior RFS suggests that adverse outcomes in *KRAS*‐mutated AML might be driven by an increased risk of relapse following remission rather than by impaired initial treatment response. These findings indicate that *KRAS* mutations may increase the risk of relapse, a hypothesis that warrants validation in larger, prospectively characterized cohorts.

MAPK pathway mutations have been reported as late events in clonal evolution, and emerging RAS mutations have been implicated in resistance to FLT3 inhibitors and BCL2 inhibitors [14, 15]. Notably, in our cohort, 12 of 24 (50%) *KRAS*‐mutated cases harbored concurrent *TET2* mutations, raising the possibility that KRAS‐driven leukemogenesis may arise on an epigenetically primed background given that *TET2* mutations are typically early clonal events [16, 17]. This epigenetically primed RAS‐driven model aligns with the concept that post‐remission survival is influenced not only by proliferative signaling but also by the underlying clonal architecture that permits rapid evolutionary adaptation. Our findings suggest that *KRAS* mutation may represent a clinically relevant prognostic factor that could contribute to improved risk stratification in future studies.

Prior studies evaluating the prognostic impact of MAPK pathway mutations in AML have reported inconsistent associations with survival outcomes. However, when MAPK pathway mutations are analyzed according to specific RAS isoforms, particularly KRAS and NRAS, our findings appear largely consistent with prior reports. For example, Ball et al. demonstrated a markedly inferior median OS in patients with *KRAS‐*mutated AML compared with those harboring *NRAS* mutations (5.2 vs. 35.2 months) [3]. Similarly, Ali et al. reported an unadjusted median OS of 5.7 months for *KRAS*‐mutated AML compared with 15.2 months for *NRAS*‐mutated AML [6]. In the present study, we observed a comparable pattern, with a median OS of 4.2 months in *KRAS‐*mutated AML and 26.6 months in *NRAS‐*mutated AML, supporting that *KRAS* and *NRAS* mutations confer clinically distinct disease phenotypes.

This study has some limitations inherent to the single‐center retrospective analysis, such as a relatively small sample size, a homogenous population, and treatment practice at a single center. The continual refinement of response assessment including MRD by NGS has also increased exponentially over time; patients treated in the earlier era may have been categorized as CR from cruder tests such as morphology and multicolor flow cytometry alone, while patients treated in the more contemporary era were more likely to be evaluated with a more sensitive NGS‐based MRD and may receive an earlier MRD‐directed salvage treatment, contributing to a longer OS. Finally, this study is subject to several potential biases, including selection bias, immortal time bias in analyses involving HSCT, and era effects reflecting evolving treatment strategies and improvements in supportive care.

In summary, in this real‐world cohort of patients with newly diagnosed MAPK‐mutated AML, *KRAS* mutations were independently associated with inferior OS and RFS despite comparable initial treatment responses. These findings suggest that the adverse prognostic impact of *KRAS* mutations may be driven by post‐remission disease biology, particularly an increased risk of relapse, rather than by treatment insensitivity. Our results underscore the need for improved post‐remission strategies and prospective studies to better define the biological and clinical implications of *KRAS* mutation in AML patients. Future studies incorporating larger cohorts and comparisons with AML lacking MAPK pathway mutations will be important to further define the prognostic and biological significance of *KRAS* mutations in AML.

## Author Contributions

K.T. and K.P. designed the study. S.M. analyzed the data. K.T., R.Y., S.S., M.T., C.W., and K.P. collected the data. K.T., S.M., M.T., and K.P. wrote the paper. K.P. supervised the study.

## Funding

The authors have nothing to report.

## Ethics Statement

This study was approved by the University of Iowa Institutional Review Board.

## Conflicts of Interest

The authors declare no conflicts of interest.

## Supporting information


**Table S1:** List of the 36 genes included in the next generation sequencing panel.
**Table S2:** Detailed MAPK pathway mutations at diagnosis identified of entire cohort (*n* = 74).
**Table S3:** The detailed next generation sequencing results of entire cohort (*n* = 74).
**Table S4:** Response to hypomethylating agent by European LeukemiaNet 2022 response criteria.
**Table S5:** Cox regression analyses of relapse‐free survival among patients achieving composite complete remission (*n* = 31).
**Table S6:** Univariable and multivariable logistic regression analyses of receiving stem cell transplantation (*n* = 62).

## Data Availability

The data that support the findings of this study are available from the corresponding author upon reasonable request.
