# Supplementary material for: KRAS Mutations Predict Inferior Post‐Remission Outcomes in Newly Diagnosed Acute Myeloid Leukemia With MAPK Pathway Mutations
Source: Eur J Haematol. 2026 Jul 24;117(5):1318–26. doi: 10.1111/ejh.70270 (PMC13643612; doi:10.1111/ejh.70270)
Supplement: Supplementary file 1 [file EJH-117-1318-s001.docx]

**Supporting information**

**Supplemental Table 1** List of the 36 genes included in the next generation sequencing panel.

| **Genes** | *ANKRD26*, *ASXL1*, *BCOR*, *BRAF*, *CBL*, *CEBPA*, *DDX41*, *DNMT3A*, *EZH2*, *FLT3*, *GATA2*, *HRAS*, *IDH1*, *IDH2*, *JAK2*, *KIT*, *KRAS*, *KMT2A* (*MLL*), *NF1*, *NPM1*, *NRAS*, *PHF6*, *PPM1D*, *PTEN*, *PTPN11*, *RUNX1*, *SETBP1*, *SF3B1*, *SRSF2*, *STAG2*, *TET2*, *TP53*, *U2AF1*, *UBA1*, *WT1*, *ZRSR2* |
| --- | --- |

**Supplemental Table 2** Detailed MAPK pathway mutations at diagnosis identified of entire cohort (n=74).

| **MAPK pathway mutations** | | | | | | | |
| --- | --- | --- | --- | --- | --- | --- | --- |
| ***NRAS*** | **Freq.** | ***KRAS*** | **Freq.** | ***BRAF*** | **Freq.** | ***HRAS*** | **Freq.** |
| G12D | 14 | G12D | 6 | V600E | 3 | H27fs | 1 |
| G13D | 10 | A146T | 3 | N581S | 1 |  |  |
| Q61H | 8 | G13D | 3 |  |  |  |  |
| Q61K | 8 | G12S | 2 |  |  |  |  |
| Q61R | 5 | G12V | 2 |  |  |  |  |
| G12C | 4 | K117N | 2 |  |  |  |  |
| G12R | 3 | A146V | 1 |  |  |  |  |
| G13R | 3 | A146P | 1 |  |  |  |  |
| G12A | 1 | G12A | 1 |  |  |  |  |
| G12S | 1 | G13C | 1 |  |  |  |  |
| G13V | 1 | G60V | 1 |  |  |  |  |
| G61L | 1 | Q61H | 1 |  |  |  |  |
|  |  | Q61P | 1 |  |  |  |  |
|  |  | Q61R | 1 |  |  |  |  |

Abbreviations: Freq, frequency.

**Supplemental Table 3** The detailed next generation sequencing results of entire cohort (n=74).

| **Patient Number** | **Next generation sequencing result** |
| --- | --- |
| 1 | *NRAS* p.G13D, *SRSF2* p.95H, *TET2* p.G1370E, *TET2* p.R1452X |
| 2 | *NRAS* p.Q61H, *TP53* p.R110L |
| 3 | *KRAS* p.G12S, *NF1* c.1709_1718+4del/insATCTATTT, *NRAS* p.G12D,  *RUNX1* p.A251Vfs*3 |
| 4 | *KRAS* p.G12D, *NPM1* p.W288fs*12 |
| 5 | *NRAS* p.G12D |
| 6 | *FLT3* p.D835E (TKD), *NRAS* p.G13R |
| 7 | *FLT3* p.D835V (TKD), *KRAS* p.G13D, *KRAS* p.G12S, *PHF6* p.C292* |
| 8 | *NRAS* p.G12D, *SRSF2* p.P95H, *TET2* p.L406Ffs*36 |
| 9 | *NRAS* p.G12C |
| 10 | *IDH2* p.F148fs, *KRAS* p.G12D |
| 11 | *FLT3* p.D835Y (TKD), *NRAS* p.G13D, *TP53* p.H214R |
| 12 | *ASXL1* p.Gly649TrpfsTer9, *KRAS* p.G12V, *RUNX1* p.Leu339ProfsTer265,  *TET2* p.Q659E, *U2AF1* p.S34F |
| 13 | *NPM1* p.W288Cfs*12, *NRAS* p.G13D, *WT1* p.C411I |
| 14 | *CEBPA* p.R35GfsTer125, *CEBPA* p.Q312_K313insNKAKQRHVETQQ  *NRAS* p.G12D, *NRAS* p.Q13H, *SETBP1* p.V231L |
| 15 | *JAK2* p.V617F *NRAS* p.G12C, *NRAS* p.G12D |
| 16 | *FLT3* p.R607_E608ins23 (ITD), *IDH2* p.R140Q, *NRAS* p.G12R, *NRAS* p.G13V |
| 17 | *DNMT3A* p.R882H, *FLT3* 36 bp duplication (ITD), *IDH2* p.R140Q, *NPM1* p.W288Cfs*12  *NRAS* p.G12D, *TET2* p.Y605* |
| 18 | *NRAS* p.G12D, *NRAS* p.G13D, *NRAS* p.Q61H |
| 19 | *KRAS* p.G13C, *NRAS* p.G13D |
| 20 | *DNMT3A* p.R882C, *IDH2* p.R140Q, *KRAS* p.G12V, *NPM1* p.W288CfsTer12  *NRAS* p.G13R |
| 21 | *IDH2* p.R140Q, *NRAS* p.Q61R |
| 22 | *BRAF* p.N581S, *GATA2* p.R307W, *TET2* p.S842Ter, *TET2* p.H1382Q, *ZRSR2* p.C181Y |
| 23 | *KRAS* p.G60V, *NF1* p.N45S, *NRAS* p.G12D, *TET2* p.Q1414L TET2 p.R1896T  *U2AF1* p.Q157R, *U2AF1* p.S34F |
| 24 | *CBL* p.C396R, *KRAS* p.Q61R, *SRSF2* p.P95L, *TET2* p.Q116*, *TET2* p.S907Ffs*15 |
| 25 | *NRAS* p.Q61H, *U2AF1* p.Q157P |
| 26 | *FLT3* p.D835Y (TKD), *NPM1* p.W290RfsTer10, *NRAS* p.Q61H, *PTPN11* p.D61Y |
| 27 | *KRAS* p.K117N, *NF1* p.T1536Nfs*7, *SRSF2* p.P95H, *TET2* p.S268Cfs*25 |
| 28 | ASXL1 p.D1012Efs*9, *KRAS* p.G13D, *NPM1* p.W288Cfs*12, *SRSF2* p.P95H  *TET2* p.C1358_L1360delinsW, *TET2* c.4182+1G>C |
| 29 | *ASXL1* p.L775Ter, *KRAS* p.A146T, *NPM1* p.W288CfsTer12, *SRSF2* p.P95H  *TET2* p.P1894R |
| 30 | *KIT* p.D816V, *NRAS* p.G12R |
| 31 | *CEBPA* p.P70Lfs*90, *CEBPA* p.R306_N307insGPQR, *GATA2* p.K324E, *NRAS* Q61K, *NRAS* G13D |
| 32 | *BRAF* p.V600E, *SRSF2* p.P95H, *TET2* p.M1164R, *TET2* p.K1308Sfs*55 |
| 33 | *DNMT3A* p.R882H, *NPM1* p.W288Cfs*12, *NRAS* p.Q61R, *SETBP1* p.S1083F |
| 34 | NRAS p.G12D, *RUNX1* p.L225_S226insR, *SRSF2* p.P95H  *TET2* p.K948Nfs*5, *TET2* p.R1465* |
| 35 | *ASXL1* p.P699Lfs*4, *NRAS* Q61K, *RUNX1* p.Q397Pfs*203 |
| 36 | *KRAS* p.G12D, *TP53* p.R175H |
| 37 | *NRAS* p.G12R, *RUNX1* p.R72Efs*66, *SRSF2* p.P95H |
| 38 | *KMT2A* p.R2211W, *NPM1* c.847-3_848delCAGGCinsATGCG, *NRAS* p.Q61R  *ZRSR2* p.S447_R448dup |
| 39 | *KIT* p.D816V, *KRAS* p.G12D |
| 40 | *KRAS* p.G12D, *TET2* p.E1144fs, *WT1* p.R370fs |
| 41 | *FLT3* ITD mutation (57 bp duplication), *KIT* p.D816Y, *KRAS* p.Q61H |
| 42 | NF1 p.P2310fs, KRAS p.Q61H |
| 43 | *KRAS* p.G12A, *NPM1* p.W288fs, *SRSF2* p.P95H *TET2* p.W1291G |
| 44 | *BRAF* p.V600E, *TET2* p.Q960* |
| 45 | *NRAS* p.Q61K, *SETBP1* p.I1871T, *SRSF2* p.P95H |
| 46 | *FLT3* *p.D835Y* (TKD mutation), *KRAS* p.Q61H |
| 47 | *JAK2* p.V617F, *KRAS* p.Q61P, *RUNX1* p.R320*, *TET2* p.Q684* |
| 48 | *BRAF* p.V600E, *DNMT3A* p.R882H |
| 49 | *ASXL1* (missing data), *NRAS* p.Q61K |
| 50 | *DNMT3A* p.P904L, *FLT3* p.D835Y (TKD mutation), *NPM1* p.W288fs, *NRAS* p.G13D |
| 51 | *IDH1* p.R132C, *NRAS* p.G12D, *U2AF1* p.S34F |
| 52 | *DNMT3A* p.R882H, *IDH1* p.R132H, *NPM1* p.W288fs, *NRAS* p.Q61R |
| 53 | *NRAS* p.G12S |
| 54 | *CEBPA* p.P239Rfs*83, *FLT3* p.V581_L610dup (ITD mutation)  *FLT3* p.E598_Y599ins11 (ITD mutation), *KRAS* p.K117N |
| 55 | *NRAS* p.Q61H |
| 56 | *IDH2* p.R140Q, *NRAS* p.Q61K |
| 57 | *NPM1* p.W288fs, *NRAS* p.Q61K |
| 58 | *KRAS* p.A146P, *SRSF2* p.P95H, *TET2* p.Y1608* |
| 59 | *NRAS* p.G13D, *NRAS* p.Q61L |
| 60 | *CEBPA* p.H18fs, *IDH1* p.R132C, *KMT2A* p.I3246T, *NRAS* p.G12D |
| 61 | *ASXL1* p.E635fs, *KRAS* p.A146T, *KRAS* p.A146V, *SF3B1* p.K700E  *TET2* c.3803+2T>G, *TET2* p.S534* |
| 62 | *DNMT3A* p.R882H, *NPM1* p.W288fs, *NRAS* p.G13D, *PTPN11* p.G60V |
| 63 | *FLT3* p.V592_D593insAGSSDNEYFYV (ITD mutation), *KMT2A* p.S2395P  *NPM1* p.W288fs, *NRAS* p.G13D |
| 64 | *JAK2* c.1777-3dupT, *KIT* p.N566S, *KRAS* p.G13D |
| 65 | *KRAS* p.G12D, *NF1* p.D1501G, *NPM1* p.W288fs |
| 66 | *IDH1* p.R132H, *NPM1* p.W288fs*12, *NRAS* p.G12D |
| 67 | *CEBPA* c.-57C>G, *HRAS* p.H27fs, *TP53* p.V203fs |
| 68 | *IDH1* p.R132C, *NRAS* p.G12D |
| 69 | *KMT2A* p.P3404T, *NRAS* p.G12D, *U2AF1* p.S34F |
| 70 | *NF1* p.T1589M, *NRAS* p.G12C, *TET2* p.S708*, *TP53* p.H179R |
| 71 | *NF1* p.S1838C, *NPM1* p.W288fs, *NRAS* p.G13R, *WT1* p.A382fs, *WT1* p.R369* |
| 72 | *FLT3* p.W603_E604insDDFREYEYDLKW (ITD mutation)  *GATA2* p.R330Q. *GATA2* p.N297S, *NRAS* p.G12A, *SF3B1* p.K700E |
| 73 | *ASXL1* p.L764fs, *KRAS* p.A146T, *NRAS* p.G12C, *SRSF2* p.P95H, *TET2* p.S1039* |
| 74 | *CBL* c.1096-1G>T, *NRAS* p.Q61K, *NRAS* p.Q61R |

**Supplemental Table 4** Response to hypomethylating agent by European LeukemiaNet 2022 response criteria.

| No. (%) | HMA  N=22 | Azacitidine-based  N=13 | Decitabine-based  N=9 |
| --- | --- | --- | --- |
| ELN 2022 response criteria | | | |
| CRc | 11 (50.0) | 4 (30.8) | 7 (77.8) |
| MLFS | 2 (9.1) | 2 (15.4) | 0 |
| PR | 1 (4.5) | 1 (7.7) | 0 |
| No response | 2 (9.1) | 0 | 2 (22.2) |
| Not evaluable | 6 (27.3) | 6 (46.1) | 0 |

Abbreviations: HMA, hypomethylating agent; ELN, European LeukemiaNet; CRc, composite complete remission; MLFS, morphologic leukemia-free state; PR, partial response.

**Supplemental Table 5** Cox regression analyses of relapse-free survival among patients achieving composite complete remission (n=31).

|  | Univariable | | | Multivariable | | |
| --- | --- | --- | --- | --- | --- | --- |
|  | HR | 95% CI | P-value | HR | 95% CI | P-value |
| Age | 1.02 | 0.99 - 1.04 | 0.21 |  |  |  |
| Sex  Female  Male | 0.80  Ref | 0.31 - 2.07 | 0.65 |  |  |  |
| Karyotype  Other  Normal | 0.73  Ref | 0.28 – 1.96 | 0.54 |  |  |  |
| ELN 2022 Risk  Adverse  Other | 1.68  Ref | 0.64 – 4.39 | 0.29 |  |  |  |
| MAPK mutation  NRAS  No  Yes  KRAS  Yes  No | 17.36  Ref  12.28  Ref | 3.19 – 94.59  2.84 – 53.02 | **<0.01**  **<0.01** | 9.51 | 2.2-41.08 | **<0.01** |
| VAF (%)  NRAS (units=1%)  KRAS (units=1%) | 1.02  1.01 | 0.99 – 1.06  0.98 – 1.04 | 0.21  0.53 |  |  |  |
| Induction treatment  Non-intensive  Intensive | 2.96  Ref | 1.08 – 8.11 | 0.04 | 2.26 | 0.79-6.48 | 0.13 |
| MRD after induction treatment  Positive  Negative | 1.06  Ref | 0.38 – 2.94 | 0.91 |  |  |  |

Abbreviations: HR, hazard ratio; CI, confidence interval; ELN, European LeukemiaNet; VAF, variant allele frequency; MRD, minimal residual disease; Ref, reference.

**Supplemental Table 6** Univariable and multivariable logistic regression analyses of receiving stem cell transplantation (n=62).

|  | Univariable | | | Multivariable | | |
| --- | --- | --- | --- | --- | --- | --- |
|  | OR | 95% CI | P-value | OR | 95% CI | P-value |
| Age | 0.92 | 0.89 - 0.96 | **<0.01** | 0.93 | 0.89 - 0.97 | **<0.01** |
| Sex  Female  Male | 0.56  Ref | 0.18 - 1.74 | 0.32 |  |  |  |
| Karyotype  Normal  Other | 1.12  Ref | 0.39 – 3.27 | 0.83 |  |  |  |
| ELN 2022 Risk  Adverse  Other | 0.37  Ref | 0.13 - 1.09 | 0.07 |  |  |  |
| MAPK mutation  NRAS  Yes  No  KRAS  Yes  No | 3.50  Ref  0.30  Ref | 0.87 - 14.03  0.09 – 1.05 | 0.08  0.06 |  |  |  |
| VAF (%)  NRAS (units=1%)  KRAS (units=1%) | 1.01  0.83 | 0.98 – 1.06  0.65 – 1.06 | 0.48  0.14 |  |  |  |
| Induction treatment  Intensive  Non-intensive | 11.05  Ref | 2.28 – 53.68 | **<0.01** | 2.15 | 0.33 – 14.04 | 0.42 |
| CRc after induction Tx  Yes  No | 2.62  Ref | 0.88 – 7.81 | 0.08 |  |  |  |

Abbreviations: OR, odds ratio; CI, confidence interval; ELN, European LeukemiaNet; VAF, variant allele frequency; CRc, composite complete remission; Tx, treatment; Ref, reference.
